# Rescuing the spike

**DOI:** 10.1186/1471-2202-14-S1-A3

**Published:** 2013-07-08

**Authors:** Sophie Denève

**Affiliations:** 1 Group for Neural Theory, LNC, DEC, ENS, 29, rue d'Ulm 75005 Paris, France

## 

Sensory and motor variables are represented by large populations of neurons. We hypothesized that these representations are constrained such that they can be read-out linearly (synaptic integration) while limiting the metabolic cost. Such framework can predict many aspects of neural tuning, robustness and adaptation. Moreover, spiking networks with balanced excitation and inhibition naturally produce such efficient codes. In such balanced networks, each membrane potential monitors the coding error, e.g. the mismatch between the feed-forward inputs and the prediction of these inputs by lateral connections. Each spike implements a greedy minimization of this error. Most importantly, any network of integrate and fire neurons will self-organize into this optimal regime with a simple Hebbian plasticity rule enforcing the balance between excitation and inhibition. Through learning, initially regular, highly correlated spike trains evolve towards Poisson-distributed, asynchronous spike trains with much lower firing rates. Importantly, single unit variability is a consequence of degeneracy in the code, not noise: the population as a whole tracks the signal perfectly. This suggests that balanced spiking networks are not equivalent to rate models, but in fact orders of magnitude more reliable.

